# Supplementary material for: MaveDB: an open-source platform to distribute and interpret data from multiplexed assays of variant effect
Source: Genome Biol. 2019 Nov 4;20:223. doi: 10.1186/s13059-019-1845-6 (PMC6827219; doi:10.1186/s13059-019-1845-6)
Supplement: Supplementary file 1 — Additional file 1: Figure S1. Data unavailability is a persistent feature of the MAVE literature. We compiled a list of 159 publications that contained at least one new deep mutational scanning or massively parallel reporter assay dataset and manually inspected the publication’s supplementary data and methods to determine whether counts or scores for the assayed variants were present. Refer to https://github.com/VariantEffect/MaveReferences for the full table. This figure was generated from release v0.1.1. Of the 159 total publications, 91 (57%) provide scores or counts. Figure S2. UML (Unified Markup Language) diagram of the complete MaveDB schema in PDF format. The diagram was generated using the Django Extensions package and visualized using Graphviz. [file 13059_2019_1845_MOESM1_ESM.pdf]

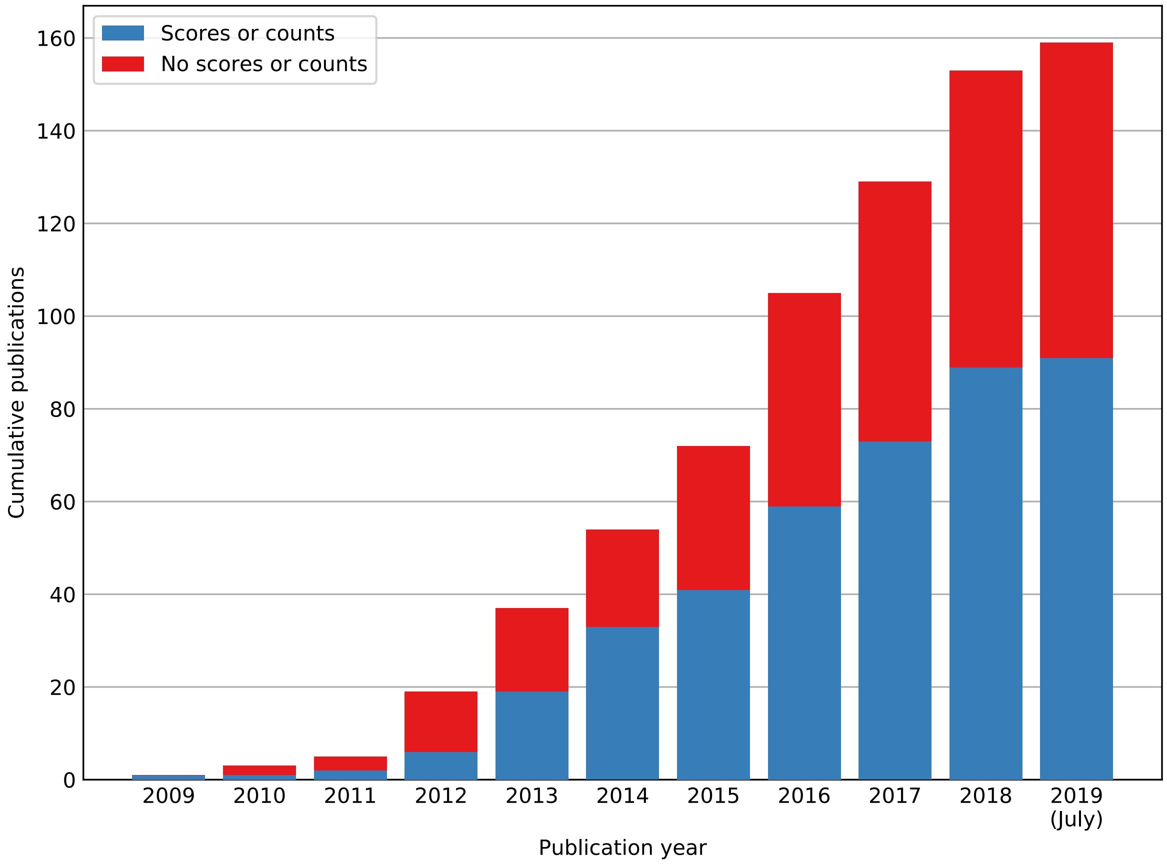

Fig S1: Data unavailability is a persistent feature of the MAVE literature. We compiled a list of 159 publications that contained at least one new deep mutational scanning or massively parallel reporter assay dataset and manually inspected the publication's supplementary data and methods to determine whether counts or scores for the assayed variants were present. Refer to https://github.com/VariantEffect/MaveReferences for the full table. This figure was generated from release v0.1.1. Of the 159 total publications, 91 (57%) provide scores or counts.

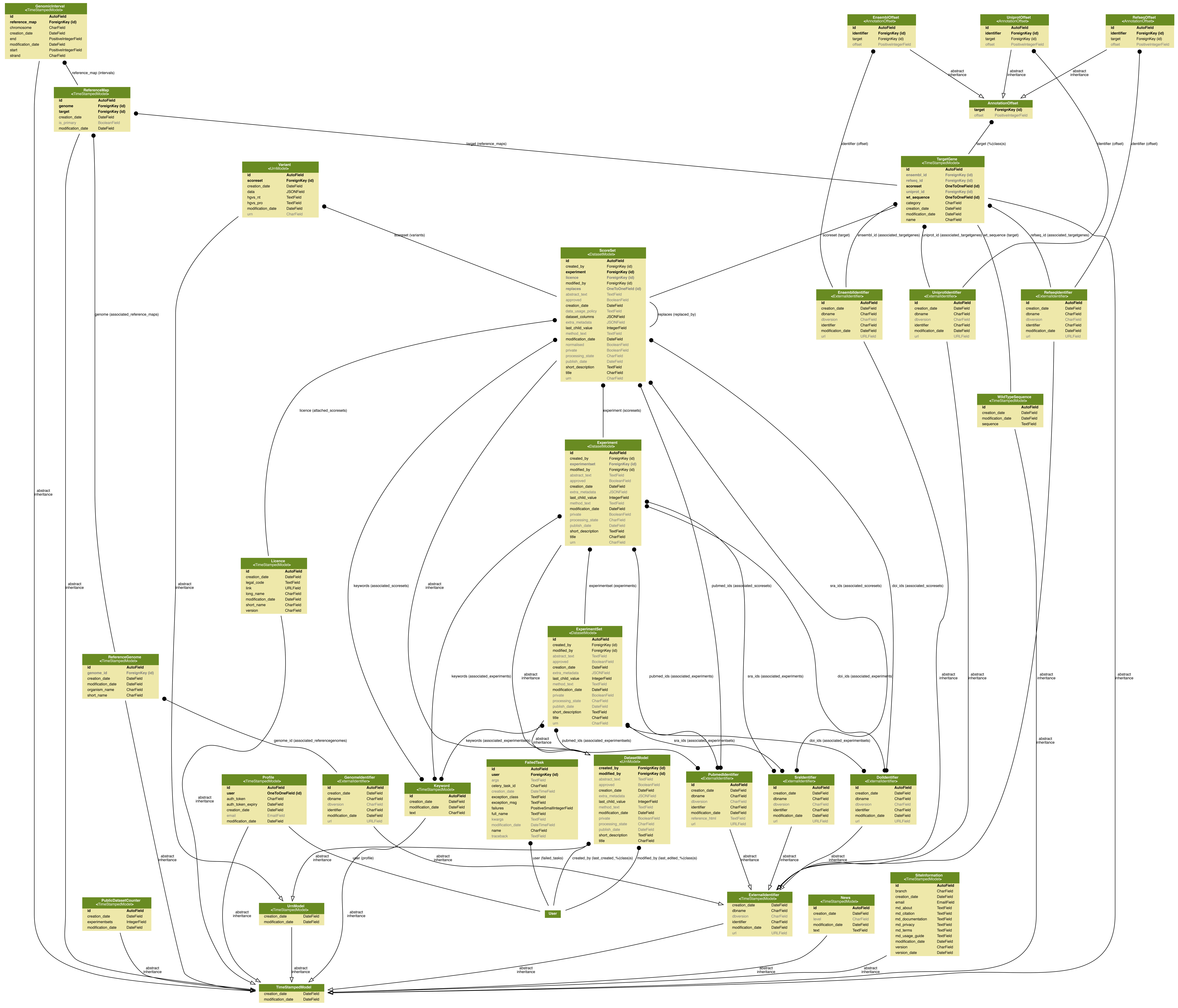
GenomicInterval
<TimeStampedModel>
id AutoField
reference_map ForeignKey (id)
chromosome CharField
creation_date DateField
end PositiveIntegerField
modification_date DateField
start PositiveIntegerField
strand CharField
reference_map (intervals)
ReferenceMap
<TimeStampedModel>
id AutoField
genome ForeignKey (id)
target ForeignKey (id)
creation_date DateField
is_primary BooleanField
modification_date DateField
target (reference_maps)
genome (associated_reference_maps)
EnsemblOffset
<AnnotationOffset>
id AutoField
identifier ForeignKey (id)
target ForeignKey (id)
offset PositiveIntegerField
UniprotOffset
<AnnotationOffset>
id AutoField
identifier ForeignKey (id)
target ForeignKey (id)
offset PositiveIntegerField
RefseqOffset
<AnnotationOffset>
id AutoField
identifier ForeignKey (id)
target ForeignKey (id)
offset PositiveIntegerField
AnnotationOffset
target ForeignKey (id)
offset PositiveIntegerField
target (%(class)s)
identifier (offset)
TargetGene
<TimeStampedModel>
id AutoField
ensembl_id ForeignKey (id)
refseq_id ForeignKey (id)
scoreset OneToOneField (id)
uniprot_id ForeignKey (id)
wt_sequence OneToOneField (id)
category CharField
creation_date DateField
modification_date DateField
name CharField
Variant
<UrnModel>
id AutoField
scoreset ForeignKey (id)
creation_date DateField
data JSONField
hgvs_nt TextField
hgvs_pro TextField
modification_date DateField
urn CharField
scoreset (variants)
scoreset (target)
ensembl_id (associated_targetgenes)
uniprot_id (associated_targetgenes)
wt_sequence (target)
refseq_id (associated_targetgenes)
ScoreSet
<DatasetModel>
id AutoField
created_by ForeignKey (id)
experiment ForeignKey (id)
licence ForeignKey (id)
modified_by ForeignKey (id)
replaces OneToOneField (id)
abstract_text TextField
approved BooleanField
creation_date DateField
data_usage_policy TextField
dataset_columns JSONField
extra_metadata JSONField
last_child_value IntegerField
method_text TextField
modification_date DateField
normalised BooleanField
private BooleanField
processing_state CharField
publish_date DateField
short_description TextField
title CharField
urn CharField
replaces (replaced_by)
EnsemblIdentifier
<ExternalIdentifier>
id AutoField
creation_date DateField
dbname CharField
dbversion CharField
identifier CharField
modification_date DateField
url URLField
UniprotIdentifier
<ExternalIdentifier>
id AutoField
creation_date DateField
dbname CharField
dbversion CharField
identifier CharField
modification_date DateField
url URLField
RefseqIdentifier
<ExternalIdentifier>
id AutoField
creation_date DateField
dbname CharField
dbversion CharField
identifier CharField
modification_date DateField
url URLField
WildTypeSequence
<TimeStampedModel>
id AutoField
creation_date DateField
modification_date DateField
sequence TextField
licence (attached_scoresets)
experiment (scoresets)
Experiment
<DatasetModel>
id AutoField
created_by ForeignKey (id)
experimentset ForeignKey (id)
modified_by ForeignKey (id)
abstract_text TextField
approved BooleanField
creation_date DateField
extra_metadata JSONField
last_child_value IntegerField
method_text TextField
modification_date DateField
private BooleanField
processing_state CharField
publish_date DateField
short_description TextField
title CharField
urn CharField
Licence
<TimeStampedModel>
id AutoField
creation_date DateField
legal_code TextField
link URLField
long_name CharField
modification_date DateField
short_name CharField
version CharField
keywords (associated_scoresets)
experimentset (experiments)
pubmed_ids (associated_scoresets)
sra_ids (associated_scoresets)
doi_ids (associated_scoresets)
ExperimentSet
<DatasetModel>
id AutoField
created_by ForeignKey (id)
modified_by ForeignKey (id)
abstract_text TextField
approved BooleanField
creation_date DateField
extra_metadata JSONField
last_child_value IntegerField
method_text TextField
modification_date DateField
private BooleanField
processing_state CharField
publish_date DateField
short_description TextField
title CharField
urn CharField
keywords (associated_experiments)
pubmed_ids (associated_experiments)
sra_ids (associated_experiments)
doi_ids (associated_experiments)
ReferenceGenome
<TimeStampedModel>
id AutoField
genome_id ForeignKey (id)
creation_date DateField
modification_date DateField
organism_name CharField
short_name CharField
genome_id (associated_referencegenomes)
keywords (associated_experimentsets)
pubmed_ids (associated_experimentsets)
sra_ids (associated_experimentsets)
doi_ids (associated_experimentsets)
Profile
<TimeStampedModel>
id AutoField
user OneToOneField (id)
auth_token CharField
auth_token_expiry DateField
creation_date DateField
email EmailField
modification_date DateField
GenomeIdentifier
<ExternalIdentifier>
id AutoField
creation_date DateField
dbname CharField
dbversion CharField
identifier CharField
modification_date DateField
url URLField
Keyword
<TimeStampedModel>
id AutoField
creation_date DateField
modification_date DateField
text CharField
FailedTask
id AutoField
user ForeignKey (id)
args TextField
celery_task_id CharField
creation_date DateTimeField
exception_class TextField
exception_msg TextField
failures PositiveSmallIntegerField
full_name TextField
kwargs TextField
modification_date DateTimeField
name CharField
traceback TextField
DatasetModel
<UrnModel>
created_by ForeignKey (id)
modified_by ForeignKey (id)
abstract_text TextField
approved BooleanField
creation_date DateField
extra_metadata JSONField
last_child_value IntegerField
method_text TextField
modification_date DateField
private BooleanField
processing_state CharField
publish_date DateField
short_description TextField
title CharField
PubmedIdentifier
<ExternalIdentifier>
id AutoField
creation_date DateField
dbname CharField
dbversion CharField
identifier CharField
modification_date DateField
reference_html TextField
url URLField
SraIdentifier
<ExternalIdentifier>
id AutoField
creation_date DateField
dbname CharField
dbversion CharField
identifier CharField
modification_date DateField
url URLField
DoiIdentifier
<ExternalIdentifier>
id AutoField
creation_date DateField
dbname CharField
dbversion CharField
identifier CharField
modification_date DateField
url URLField
user (profile)
user (failed_tasks)
created_by (last_created_%(class)s)
modified_by (last_edited_%(class)s)
User
UrnModel
<TimeStampedModel>
creation_date DateField
modification_date DateField
ExternalIdentifier
<TimeStampedModel>
creation_date DateField
dbname CharField
dbversion CharField
identifier CharField
modification_date DateField
url URLField
News
<TimeStampedModel>
id AutoField
creation_date DateField
level CharField
modification_date DateField
text TextField
SiteInformation
<TimeStampedModel>
id AutoField
branch CharField
creation_date DateField
email EmailField
md_about TextField
md_citation TextField
md_documentation TextField
md_privacy TextField
md_terms TextField
md_usage_guide TextField
modification_date DateField
version CharField
version_date DateField
PublicDatasetCounter
<TimeStampedModel>
id AutoField
creation_date DateField
experimentsets IntegerField
modification_date DateField
abstract
inheritance
TimeStampedModel
creation_date DateField
modification_date DateField

Fig S2: UML (Unified Markup Language) diagram of the complete MaveDB schema in PDF format. The diagram was generated using the Django Extensions package and visualized using Graphviz.
