# Supplementary material for: MaveDB: an open-source platform to distribute and interpret data from multiplexed assays of variant effect
Source: Genome Biol. 2019 Nov 4;20:223. doi: 10.1186/s13059-019-1845-6 (PMC6827219; doi:10.1186/s13059-019-1845-6)
Supplement: Supplementary file 2 — Additional file 2. Review history. [file 13059_2019_1845_MOESM2_ESM.docx]

Review History

**First round of review**

**Reviewer 1**

The functional consequences of missense mutations on protein function are rarely known. This impedes the interpretation of clinical data leading to a growing list of variants of uncertain significance. To democratize the effort of variant interpretation this study describes a new database to provide the community with a resource to mine multiplex assays of variant effects (MAVE). These studies systematically assess the functional impact of mutations by converting every amino acids of a polypeptide chain into the remaining 19 amino acids. To analyse protein coding sequences the experimental readout is typically the survival of the cells where the mutant proteins are expressed or the cellular abundance. 
The effort is timely, relevant and of great value to scientists and clinicians interested to understand the sequence-structure-function relationship of protein and enhancer function. I am in support of publication of this 'database issue' in Genome Biology if the below points are clarified. I felt there are a few technical glitches affecting user friendliness that should be addressed.


1)     Does the database focus on missense mutations or truncating mutations of splice site mutations covered as well?
2)     Does the database solely cover instances where mutations are tested 'one-by-one' or are studies that test for combinatorial (non-additive) mutations also considered. Do authors feel that testing mutations individually is the only path forward or should combinatorial (epistatic) effects (by randomizing 2, 3 or 4 sites at once) also be considered? 
3)     I find Figure 1 confusing. The main text explains where scores sets and experiments could be derived from (replicates, different assays for the same sequence set). The figure would be more informative if it is self-explanatory.
4)     The authors scrutinized 155 publications with MAVE type data but only 50% include score sets. I believe only data with score sets end up in the searchable database. I recommend a supplementary table that includes all 155 studies otherwise part of the effort made by the authors is lost.
5)     Currently the database contains 45 entries which is less than the 50% of the 155 studies of which the authors state score sets are available.
6)     I didn't find it easy to switch between mavedb.org and vis.varianteffect.org. mavedb is browsable but I could not identify a button that takes me directly to the graphical visualization of variant effects.
7)     I have tried to visualize BRCA1 and PTEN data on vis.varianteffect.org but the 'visualise' button was not activatable.  I could only inspect the example.
8)     If would find it useful if MaveVis could highlight natural or disease associate mutations from databases such as ClinVar, COSMIC or GnomAD?
9)     In maveVis heatmaps are yellow fields missing scores?
10)      It would be useful if in future developments 'mouse over' heatmaps would display detailed mutation data.

**Reviewer 2**

Having a centralized database/portal that summarizes and provides organized access to the MAVE or other functional screening data is an excellent idea. 
However, neither the manuscript, nor the database itself is of enough scientific value to benefit researchers downstream at the moment.

Overall the manuscript is not written in scientific way with very sketch figures and descriptions that are more suitable for an online help/manual.
There are a lot of scientific content missing,
particularly the metadata (context) under which the experiments were performed are poorly described/captured.  Missing these information makes it impossible to reuse the data in further meta-analysis.
Variants can have very different functions in different organisms, isoforms, model organisms (e.g., different species, genetic background, cell-lines, etc.).  
The work also does not provide info to help better understand/interpret the meaning of the functional scores (beyond what they were in the original publication).  This would have been an important contribution as the scores from different experiments/platforms are highly heterogeneous.

In addition, the volume of the data appeared actually small (at least for homo sapien).    

-"As a consequence, the total number of variants with functional data generated by 43 MAVEs was predicted to surpass 200,000 by the end of 2018 [3], which exceeds the number of classified 44 missense variants available in ClinVar [5]."
Are these all human data?  If not, why comparing with ClinVar?  Also functional screening is not equivalent to clinical significance.  Not sure comparing these numbers makes sense.

-"MaveDB currently contains over one million variant effect scores across 39 unique targets"
Citation to this?  Does this 39 targets refer to 39 genes?  That sounds very small.

In summary, I think it is a great idea to develop such a portal.  However, the current status of the database and the design do not seem to facilitate necessary advances towards creating a valuable resources for the research community.  The manuscript needs to significantly rewritten for biological readers and provide biologically interesting content instead of technical/implementational details.

**Authors Response**

**Point-by-point responses to the reviewers’ comments:**

Responses to Reviewer 1:

The functional consequences of missense mutations on protein function are rarely known. This impedes the interpretation of clinical data leading to a growing list of variants of uncertain significance. To democratize the effort of variant interpretation this study describes a new database to provide the community with a resource to mine multiplex assays of variant effects (MAVE). These studies systematically assess the functional impact of mutations by converting every amino acids of a polypeptide chain into the remaining 19 amino acids. To analyse protein coding sequences the experimental readout is typically the survival of the cells where the mutant proteins are expressed or the cellular abundance. 
>The effort is timely, relevant and of great value to scientists and clinicians interested to understand the sequence-structure-function relationship of protein and enhancer function. I am in support of publication of this 'database issue' in Genome Biology if the below points are clarified. I felt there are a few technical glitches affecting user friendliness that should be addressed. 

*We appreciate this thoughtful and constructive summary, and hope that we have addressed the issues raised below.* 

1) Does the database focus on missense mutations or truncating mutations of splice site mutations covered as well? 

*While most experiments performed so far have focused primarily on missense, nonsense, and synonymous variants, MaveDB also supports other mutations that can be described in HGVS format, including splice-site variants. We now make this point and the following point more clearly in the “Construction and content” section (lines 102-103).* 

2) Does the database solely cover instances where mutations are tested 'one-by-one' or are studies that test for combinatorial (non-additive) mutations also considered. Do authors feel that testing mutations individually is the only path forward or should combinatorial (epistatic) effects (by randomizing 2, 3 or 4 sites at once) also be considered? 

*MaveDB does support describing the effects of combinatorial mutations occurring both in cis and in trans via HGVS syntax. Indeed many datasets, such as the phage display results for the Yap65 WW domain already contain double and triple mutant entries, for example p.[Gln26Pro;Pro33Ala] and p.[Lys12Asn;Arg18Pro;Phe20Tyr] (see https://www.mavedb.org/scoreset/urn:mavedb:00000002-a-1/). Overall, more than half of the variants in the database are multi-mutants (although we note that these are not uniformly distributed across datasets). We are strongly in support of describing multi-mutant behavior, as genetic interactions can identify functional relationships and illuminate secondary and tertiary structures.* 

3) I find Figure 1 confusing. The main text explains where scores sets and experiments could be derived from (replicates, different assays for the same sequence set). The figure would be more informative if it is self-explanatory. 
 *We have redesigned Figure 1 to show how two real datasets are structured in MaveDB (see Figure 1 and the new legend, lines 678-683). We hope that readers will now find the figure to be much more understandable and engaging.* 

4) The authors scrutinized 155 publications with MAVE type data but only 50% include score sets. I believe only data with score sets end up in the searchable database. I recommend a supplementary table that includes all 155 studies otherwise part of the effort made by the authors is lost. 

*We agree that a table presenting all the studies would be a valuable resource for researchers in this field. However, as a supplemental table it would become out-of-date rapidly and be difficult to update as datasets from these studies become available online. To address this, we have released the table of studies on GitHub at the following URL: https://github.com/VariantEffect/MaveReferences 

This new resource is referenced in the figure legend as the source of data for Supplemental Figure 1 (lines 699-701) and is also included in the “Availability of data and materials” section (line 218). 

The repository is part of the VariantEffect ‘organization’ within GitHub, which already hosts the source code for MaveDB, MaveVis, and related tools. We hope that making the table available in a durable but editable form will allow it to remain up to date, and other members of the community can contribute to or amend the table using standard GitHub collaborative tools. 

Since our original submission, we have also added four additional studies (for a total of 159), updated existing references (reflecting the peer-reviewed publication of preprints), and modified Supplemental Figure 1 and its legend accordingly (lines 696-701).* 

5) Currently the database contains 45 entries which is less than the 50% of the 155 studies of which the authors state score sets are available. 

*We have reviewed the list of studies and uploaded eight additional datasets (from six published studies) that we have obtained permission to include. We’ve contacted the authors of the remaining studies and asked for permission to deposit their data, which we anticipate doing over the coming months. We now present 15 of the 91 studies that provided scores and are therefore theoretically able to be deposited in MaveDB, as well as scores from three published studies for which scores were not previously available and two unpublished datasets. This adds up to a total of 55 datasets at present. 

We have not yet included studies that provided only counts or raw sequencing reads, because this would require us to re-implement the computational analysis pipeline for each paper. Neither have we included studies where no data are provided. We have also contacted the corresponding authors of these studies offering to assist their labs in creating MaveDB entries for their datasets. 

We hope the additional legitimacy that will be imparted to MaveDB via its publication, together with the critical mass of data that we have already entered, will encourage others to contact us or upload their previously published data themselves. We have accelerated this by contacting authors of previous studies, and have already received several encouraging responses. 

Going forward, we hope that journals will require data deposition, and believe that MaveDB has an excellent chance to become the ‘database of record’ for MAVEs. In this case, the need for us to perform additional manual entry would be limited largely to datasets that were published before most journals make data deposition mandatory.* 

6) I didn't find it easy to switch between mavedb.org and vis.varianteffect.org. mavedb is browsable but I could not identify a button that takes me directly to the graphical visualization of variant effects. 

*Thank you for this comment. We have moved the button that links each scoreset to MaveVis to a more prominent location. 

We have also revised the page layout to emphasize the title and description rather than the accession number, and added score set links near the top of each experiment page so that users can get to the data and visualizations more easily. Each of these links displays the score set’s title and description as a tooltip when moused over. 

We hope that these changes will make the resource more intuitive and easier to use.* 

7) I have tried to visualize BRCA1 and PTEN data on vis.varianteffect.org but the 'visualise' button was not activatable. I could only inspect the example. 

*Thank you for this helpful feedback. We tested and confirmed that both of these datasets work, but they required that both a Uniprot ID and at least one PDB ID provided by the user. To avoid future confusion about these requirements we decided to make the structure and conservation tracks in the visualization optional, so that these inputs can be omitted if desired. We have added cues to the interface that let the user know that adding these IDs will allow them to have added structure and conservation tracks. 

In addition, to improve the user experience, we have updated MaveDB to only enable the MaveVis button for score sets that contain amino acid variant data, which MaveVis requires for visualization.* 

8) If would find it useful if MaveVis could highlight natural or disease associate mutations from databases such as ClinVar, COSMIC or GnomAD? 

*Thank you for this comment. This is indeed one of several planned features for a future update of MaveVis, but we did not want to delay dissemination of MaveDB until this addition could be made in a robust fashion.* 

9) In maveVis heatmaps are yellow fields missing scores? 

*The yellow fields represent the wild type amino acid at each position. While this is indicated in the legend on the right hand side of each generated image, we realized that for larger maps, the legend is usually so far to the right that it ends up off-screen, and only visible when users scroll all the way to the right edge. To alleviate this problem, we have added an additional legend to be displayed below the data input panel, where it remains in view at all times.* 

10) It would be useful if in future developments 'mouse over' heatmaps would display detailed mutation data. 

*Thank you for this comment. We have now included this feature. A tooltip is displayed when hovering the mouse over elements of the map. This includes the score (and error estimate where available) for each variant, as well as summary statistics for the annotation tracks.*

Responses to Reviewer 2:

Having a centralized database/portal that summarizes and provides organized access to the MAVE or other functional screening data is an excellent idea. 
However, neither the manuscript, nor the database itself is of enough scientific value to benefit researchers downstream at the moment. 
Overall the manuscript is not written in scientific way with very sketch figures and descriptions that are more suitable for an online help/manual. 

*We hope that, as suggested by the Editor, the more explicit classification of the revised manuscript as a Database article now provides sufficient clarity to the reader that the goals and scope of our report are primarily to provide a useful repository and API for variant effect data.* 
 *We would be happy to address any specific issues with the figures or descriptions, but in the absence of detail we can only hope that the changes we have made to the revised manuscript have addressed whatever the specific concerns might have been.* 

1) There are a lot of scientific content missing, particularly the metadata (context) under which the experiments were performed are poorly described/captured. Missing these information makes it impossible to reuse the data in further meta-analysis. 
Variants can have very different functions in different organisms, isoforms, model organisms (e.g., different species, genetic background, cell-lines, etc.). 

*We completely agree that metadata is important. As we are establishing a new database for a new datatype, however, it is essential that barriers for data upload are initially minimal. It is worth considering that many microarray studies were published in the years before the MIAME standards for inclusion of metadata (Brazma et al., Nature Genetics, 2001), and yet the datasets preceding establishment of these standards still merited capture within standardized databases. 

Regarding organism and isoform, we do strictly require that users upload this information as part of defining the target sequence. 

More granular information about the precise growth conditions, genetic background etc. is of course also important metadata. We provide fields that data providers can use to record such data, and all of these metadata fields are fully searchable and accessible via the API. Unfortunately, we are unable to include comprehensive methods for published studies without the risk of infringing on journal copyright, but we have gone back through the datasets we contributed and populated the Abstract and Methods fields with relevant details to improve discoverability and provide context. Each dataset also now has multiple keywords that capture features of the experimental system and methods, such as the mutagenesis type and sequencing method. 

We have also added columns containing some of these details to our supplemental table of MAVE studies (available at https://github.com/VariantEffect/MaveReferences), and hope that this will be a useful* resource for future meta-analyses. 

*When, as we expect, metadata standards exist that are widely accepted by data providers and journals, we will be better placed to require these fields and enforce said standards. We hope that users who are depositing unpublished data will make use of the Methods metadata fields to provide detailed methods descriptions with details of wet-lab and dry-lab protocols, and use the Abstract metadata field to provide an interpretation of their findings. We also note that MaveDB can store associated DOIs for each entry, and that this can be used to link to detailed protocols managed by complementary services like protocols.io and Protocol Exchange.* 

2) The work also does not provide info to help better understand/interpret the meaning of the functional scores (beyond what they were in the original publication). This would have been an important contribution as the scores from different experiments/platforms are highly heterogeneous. 

*We agree that scores generated using different experimental designs and analysis approaches are heterogeneous. Since each dataset should therefore be interpreted by experts, we think this is best left to study authors and we provide publication-specific metadata so that the references for these interpretations are easily discoverable by MaveDB users. We do not feel it would be appropriate to re-interpret the datasets that we are making available, especially when in many cases we were not involved in their design or creation. 

We hope that providing variant effect data in a standardized format will make it possible for other researchers to re-interpret published datasets and perform targeted meta-analysis that can thoroughly explore these issues.* 

3) In addition, the volume of the data appeared actually small (at least for homo sapien). 

*Whether the current data volume is large or small is of course subjective, but we do capture a substantial fraction of the rapidly-growing collection of published variant effect mapping data. Indeed, we have now included all of the datasets for which variant scores and permission from the authors were available, and we have approached authors of published MAVEs in order to continue to grow the resource. 

We also note that, of the 47 datasets in the database at the time of submission, 41 had human target sequences and that these datasets amounted to a total of 992,220 variant effect measurements. We now include 55 datasets, of which 43 have human target sequences. We think it would be fair to describe the current database content as focused on Homo sapiens, although of course the generation and inclusion of additional human MAVEs will further improve the situation.* 

- "As a consequence, the total number of variants with functional data generated by 43 MAVEs was predicted to surpass 200,000 by the end of 2018 [3], which exceeds the number of classified 44 missense variants available in ClinVar [5]." -- Are these all human data? If not, why comparing with ClinVar? Also functional screening is not equivalent to clinical significance. Not sure comparing these numbers makes sense. 

*The goal of this comparison is to give the reader, who may be less familiar with MAVE data and more familiar with widely-used databases such as ClinVar, a sense of scale. While we agree that this type of functional data is not equivalent to clinical data, readers may still find the scale comparison useful. We have rewritten this sentence to try to make this point clearer (lines 42-44).* 

 -"MaveDB currently contains over one million variant effect scores across 39 unique targets" -- Citation to this? 
 *The current manuscript will be the first peer-reviewed publication describing MaveDB, so there is no external citation. The live list of all targets in MaveDB is available by API request (see https://www.mavedb.org/api/target/). This list was sorted by target name to determine uniqueness. The number of variant effect scores was determined by counting how many variants were present in the database table. 

The database now includes data on 45 unique targets, and we have updated the text accordingly (line 88).* 

- Does this 39 targets refer to 39 genes? That sounds very small. 

*Yes, with the clarification that some targets are annotated regulatory regions such as promoters or enhancers. While this is only a small number of the total number of genes that could be used as a target in a MAVE experiment, this does represent a substantial fraction of the datasets that are currently available. Furthermore, as each of these studies contains thousands or tens of thousands of variant effect measurements, we feel this does add up to a large amount of data. 

Most importantly, the number of MAVE studies continues to grow, and it seems sensible to develop and raise awareness of a repository to capture new data types before the expected tidal wave of data has arrived.* 

In summary, I think it is a great idea to develop such a portal. However, the current status of the database and the design do not seem to facilitate necessary advances towards creating a valuable resources for the research community. The manuscript needs to significantly rewritten for biological readers and provide biologically interesting content instead of technical/implementational details. 
 *We hope that, as suggested by the Editor, the more explicit classification of the revised manuscript as a Database article will make more clear to readers that, while our resource will facilitate many downstream analyses of the growing body of MAVE data from diverse studies, the core aims of this manuscript are to describe a useful repository and API for MAVE data and demonstrate an example application in MaveVis that we hope will excite biological readers and inspire future software development. 

As it is, to our knowledge, the only such repository, we believe MaveDB has a good chance to become the primary database for this data type in the future, serving as the foundation for additional applications that will support a diverse group of researchers.*

**Second round of review**

**Reviewer 1**

The authors have addressed my comments in full. I find the updated database much improved and find it easy and convenient to navigate the available datasets. I appreciate the valuable contribution to the community.

**Reviewer 2**

Authors did a great job at answer my critiques.  Now I feel appropriate to publish the work as a "Database".

Please make an effort to improve the figures and legends.

Fig 1b did not clearly express "two distinct targets". 
Figure legends need to describe every labels/abbreviations on the figure.  For example, 
in Fig. 1A, do the "-1" and "-2" suffixes represent technical replica?
in Fig. 2, what do "stop", "syn", "hyper" mean?

**Authors Response**

**Point-by-point responses to the reviewers’ comments:**

Responses to Reviewer 2:

Please make an effort to improve the figures and legends.

Fig 1b did not clearly express "two distinct targets". 
Figure legends need to describe every labels/abbreviations on the figure.  For example, 
in Fig. 1A, do the "-1" and "-2" suffixes represent technical replica?
in Fig. 2, what do "stop", "syn", "hyper" mean?

*we have updated Fig. 1 to include gene names under the DNA icons to better show that Fig. 1b is describing experiments on two distinct targets. We added text to the Fig. 1 legend describing the accession number format, including the meaning of the "-1" and "-2" suffixes. We also updated the legend of Fig 2. to include the missing definitions of "stop" "syn" and "hyper" in the heatmap color bar.*
